# Detection and characterization of spacer integration intermediates in type I-E CRISPR–Cas system

**DOI:** 10.1093/nar/gku510

**Published:** 2014-06-11

**Authors:** Zihni Arslan, Veronica Hermanns, Reinhild Wurm, Rolf Wagner, Ümit Pul

**Affiliations:** Institut für Physikalische Biologie, Heinrich-Heine-Universität, Universitätsstraße 1, 40225 Düsseldorf, Germany

## Abstract

The adaptation against foreign nucleic acids by the CRISPR–Cas system (Clustered Regularly Interspaced Short Palindromic Repeats and CRISPR-associated proteins) depends on the insertion of foreign nucleic acid-derived sequences into the CRISPR array as novel spacers by still unknown mechanism. We identified and characterized in *Escherichia coli* intermediate states of spacer integration and mapped the integration site at the chromosomal CRISPR array *in vivo*. The results show that the insertion of new spacers occurs by site-specific nicking at both strands of the leader proximal repeat in a staggered way and is accompanied by joining of the resulting 5′-ends of the repeat strands with the 3′-ends of the incoming spacer. This concerted cleavage-ligation reaction depends on the metal-binding center of Cas1 protein and requires the presence of Cas2. By acquisition assays using plasmid-located CRISPR array with mutated repeat sequences, we demonstrate that the primary sequence of the first repeat is crucial for cleavage of the CRISPR array and the ligation of new spacer DNA.

## INTRODUCTION

Clustered Regularly Interspaced Short Palindromic Repeats (CRISPR) and CRISPR-associated (Cas) proteins constitute an adaptive prokaryotic defense system against foreign genetic elements, like phages or plasmids (1–3). A typical CRISPR array consists of short repetitive sequences (repeats), which flank foreign DNA-derived variable spacer sequences of similar length (4). The adaptation against an invader and its nucleolytic destruction occurs in three successive stages, starting with the integration of short DNA pieces of the invader DNA into the CRISPR array by unknown mechanisms (adaptation stage) (2). Transcription of the repeat-spacer unit results in the formation of a long pre-crRNA, which gets processed at the repeat-spacer boundaries by Cas proteins, releasing the individual spacer sequences in form of small crRNAs (transcription/processing stage). Associated with specific Cas protein(s), the crRNAs enable the recognition of the target DNA by base-pair complementarity (interference stage) (3). Hybridization of the crRNA sequence with the complementary strand of the invader DNA leads to the formation of an R-loop structure that initiates the nucleolytic destruction of the targeted DNA (5,6). Currently, more than 10 different CRISPR–Cas subtypes are identified, which have been classified into three major types, according to their *cas* gene composition, repeat sequences and mechanistic variations in crRNA maturation and target inactivation (7–9). With the exception of type III-B subtypes that act on RNAs (10–13), the primary target of the CRISPR–Cas pathway is double-stranded DNA (3,14,15).

Recent studies have shed light into the process of the adaptation, which is still the least understood stage of the CRISPR–Cas pathway (16–20). Usually, the repeat-spacer clusters are preceded by an AT-rich leader region that harbors the promoter for transcription of the array (21,22). It was consistently reported that the incorporation of new spacer occurs immediately next to the leader, pointing to a direct involvement of leader sequences in spacer uptake (18,23–25). The acquisition of new spacer DNA is best studied for the type I-E CRISPR–Cas system of *Escherichia coli* K12 (26,27). It has been shown that Cas1 and Cas2 are sufficient to mediate the uptake of new spacer DNA into an existing CRISPR array consisting of the leader DNA that precedes at least one repeat sequence (23). *In vitro* analyses revealed that Cas1 is a metal-dependent nuclease, capable to cleave double-stranded and single-stranded DNA in a sequence-unspecific manner (28,29). On the other hand, some Cas1 proteins are also capable to cleave RNAs, whereas others do not possess any nuclease activity (30). Likewise, several Cas2 proteins have been identified as RNases with a ferrodoxin-like fold (31), while Cas2 from *Bacillus halodurans* is a metal-dependent double-stranded DNase (32). In contrast, no nuclease activity could be detected for Cas2 from *Desulfovibrio vulgaris* (33). At present, it is unclear, at which step of the adaptation pathway Cas1 and Cas2 are involved, e.g. whether they are mediating the production of spacer precursors (through cleavage of the invading DNA), or whether they are required for the opening of the CRISPR array and/or integration of spacers. In addition to Cas1 and Cas2, some CRISPR–Cas subtypes require the activity of Cas4 or Csn2 to acquire new spacers (1,15,18). Both proteins adopt a toroidal structure (34–36) and are involved in DNA-end metabolism (37,38). Indeed, Cas4 has been shown to form a higher-order protein complex with Cas1/Cas2, termed Cascis (CRISPR-associated complex for the integration of spacers) (39).

Besides these adaptation proteins, the presence of Cascade complex and Cas3 protein has been shown to promote the integration of new spacer from an invader that is already targeted by pre-existing spacers. This process has been termed ‘primed acquisition’ (24,40). In contrast to type I-E system of *E. coli*, which is able to acquire new spacers also from ‘unprimed’ targets, spacer acquisition in type I-B of *Haloarcula hispanica* has been shown to strictly depend on the presence of spacers with some sequence identity to the invading DNA and Cas proteins required for the interference stage (18). It appears that there are at least two different pathways for selection and generation of spacer precursors, one that allows a *de novo* adaptation (unprimed) and another that couples the interference stage with adaptation (primed).

The fact that in type I-E CRISPR–Cas system of *E. coli* the addition of new spacer DNA is linked to the duplication of the leader proximal repeat sequence suggests that both strands of the first repeat serve as templates for polymerase reaction in which each newly inserted spacer gets flanked by two identical repeat sequences (41). At the molecular level, the incorporation of spacers with subsequent repeat duplication could be achieved by a staggered cut of the first repeat sequence, i.e. single nicks at the 5′- or 3′-termini of the first repeat on both strands. Here, we provide the first experimental evidence for a Cas1 and Cas2-dependent staggered cleavage at the leader proximal repeat and ligation of new spacer DNA in-between the single-stranded repeat overhangs by a concerted cleavage-ligation reaction. The appearance of these intermediates depends not only on the previously identified catalytic center of Cas1 and the presence of Cas2 protein, but also on the sequence of the leader proximal repeat. The structural nature of the integration intermediates suggests an integrase activity, depending on the presence of both Cas1 and Cas2.

## MATERIALS AND METHODS

### Bacterial strains, plasmids and oligonucleotides

Strains, plasmids and sequences of oligonucleotides used in this study are listed in Supplementary Tables S3–S5. The construction of the plasmids pCR001, pCR002, pCR003WT, pCR003RM1 and pCR003RM2 are described in Supplementary Table S3.

### Spacer acquisition assays

The acquisition of spacer sequences were tested as described before (23). In brief, 100 ml YT (yeast extract tryptone) medium with or without 0.1 mM IPTG and 0.2% arabinose were inoculated with overnight cultures of BL21-AI/puC18Kan transformed either with pCR001, pCR002, pCR003WT, pCR003RM1 or pCR003RM2 (Supplementary Table S3 and S5). After growth for 18–22 h at 37°C aliquots of the cultures were diluted 1:50 and 1:500 in YT medium, and 5 μl of each were used as templates in polymerase chain reaction (PCR). Primer pairs 10 and 15 or 16 and 17 (Supplementary Table S4) were used to test an expansion of the genomic or plasmid-located CRISPR array, respectively. The absence of spacer acquisition into the plasmids pCR003RM1 and pCR003RM2 was controlled by three independent acquisition assays and PCR analyses of at least three cycles for each culture. The PCR products were analyzed on 1.2% agarose gels.

To isolate single copies of expanded CRISPR arrays for sequencing, aliquots of the liquid cultures were spread onto selective YT-agar plates, and individual clones were picked and used as templates in single colony PCR. Fragments with expanded genomic CRISPR array were purified with PCR purification Kit (Qiagen) and sent to DNA sequencing (StarSEQ, Germany). To obtain single copies of expanded pCR003 plasmid variants (see Supplementary Figure S7) total plasmid DNA of single colonies was prepared (Qiagen Mini Kit) and transformed into *E. coli* XL1 or TOP10 cells. Clones with uniform pCR003 variants were identified by a second round of single colony PCR as described above.

### Preparation and fragmentation of genomic DNA

To isolate genomic DNAs for the Southern blot analyses, two or four 10 ml aliquots of uninduced or induced cells, respectively, were harvested by centrifugation for 10 min at 6000 g. Each pellet was resuspended in 5 ml lysis buffer (10 ml of 1xPBS buffer (137 mM NaCl, 10 mM Na_2_HPO_4_, 2 mM KH_2_PO_4_, 2.7 mM KCl, pH 7.4), supplemented with 20 mg lysozyme, 10 mg proteinase K) and incubated for 45 min at 37°C. 500 μl of 10% sodium dodecyl sulphate (SDS) was added and the mixtures were incubated for additional 45 min at 37°C. After extraction with phenol/chloroform and precipitation with ethanol, the pellets were dissolved in 1 ml TE (Tris-EDTA) buffer (10 mM Tris-HCl pH 7.5, 1 mM EDTA). Ten microliters of RNase A [10 mg/ml] were added and the mixtures were incubated at 37°C for 1 h. The samples were again extracted with phenol/chloroform, precipitated with ethanol and the pellets were dissolved in 200–400 μl TE buffer.

Genomic DNA samples were digested with 1 unit of DraI (Fermentas) or BanI (NEB) per μg DNA in buffers provided by the manufacturer. The mixtures were incubated overnight at 37°C, followed by heat-deactivation of the enzymes for 25 min at 65°C.

### Southern blot analyses

Indicated amounts of genomic DNA fragments were separated either on 0.7% agarose or 10% denaturing polyacrylamide gels by electrophoresis. DNA fragments in the agarose gels were denatured by shaking of the gel in denaturation solution 1 (0.5 M NaOH/1.5 M NaCl) and in denaturation solution 2 (3 M NaOH, 0.5 M Tris-HCl, pH 7.0), each for 30 min at room temperature. The DNA fragments were blotted onto Hybond™-N+ membranes (GE Healthcare, Freiburg, Germany) either overnight by capillary transfer (from agarose gels) or by electrotransfer for 60 min at 400 mA (from denaturing polyacrylamide gels) using semi-dry blotting system (Owl Semi-Dry Blotter, Thermo Scientific). After ultraviolet (UV) crosslinking (UV Stratalinker 1800, Stratagene) the membranes were baked for 2 h at 80°C, and hybridized against 5′-^32^P-end labeled oligonucleotides overnight at appropriate temperatures (see Supplementary Table S4). The bands were visualized by autoradiography for 4 to 7 days using intensifier screens.

## RESULTS AND DISCUSSION

### Detection of spacer acquisition intermediates by Southern analyses

To study the mechanism of spacer integration into the CRISPR array *in vivo*, we adopted the assay developed by Yosef *et al*. (23). To this aim we constructed the pCR001 plasmid containing the *cas1–cas2* genes under the control of an IPTG-inducible promoter and transformed *E. coli* BL21-AI strain. Induction of the plasmid-borne *cas1–cas2* genes followed by growth of the cells for 18 h initiated the integration of new spacer DNA into the chromosomal CRISPR array (Supplementary Figure S1). The acquisition process led to an expansion of the CRISPR array by 61 bp per added spacer-repeat unit (29 bp repeat and 32 bp spacer), which can be detected by colony PCR with primers for the amplification of the CRISPR array (23) (Supplementary Figure S2). The integrated spacers were derived either from the genomic DNA or from plasmid DNA, and insertion of up to three spacers into a single CRISPR array could be observed (Supplementary Table S1). Note that *E. coli* BL21-AI strain harbors a CRISPR array (leader DNA followed by 13 repeat-spacer units), but lacks all *cas* genes. Therefore, even if plasmid-based overexpression of *cas1–cas2* provokes the integration of spacers that are originated from the host genome, a self-targeting does not take place due to the absence of the Cascade complex and Cas3 (6,42).

**Figure 1.**
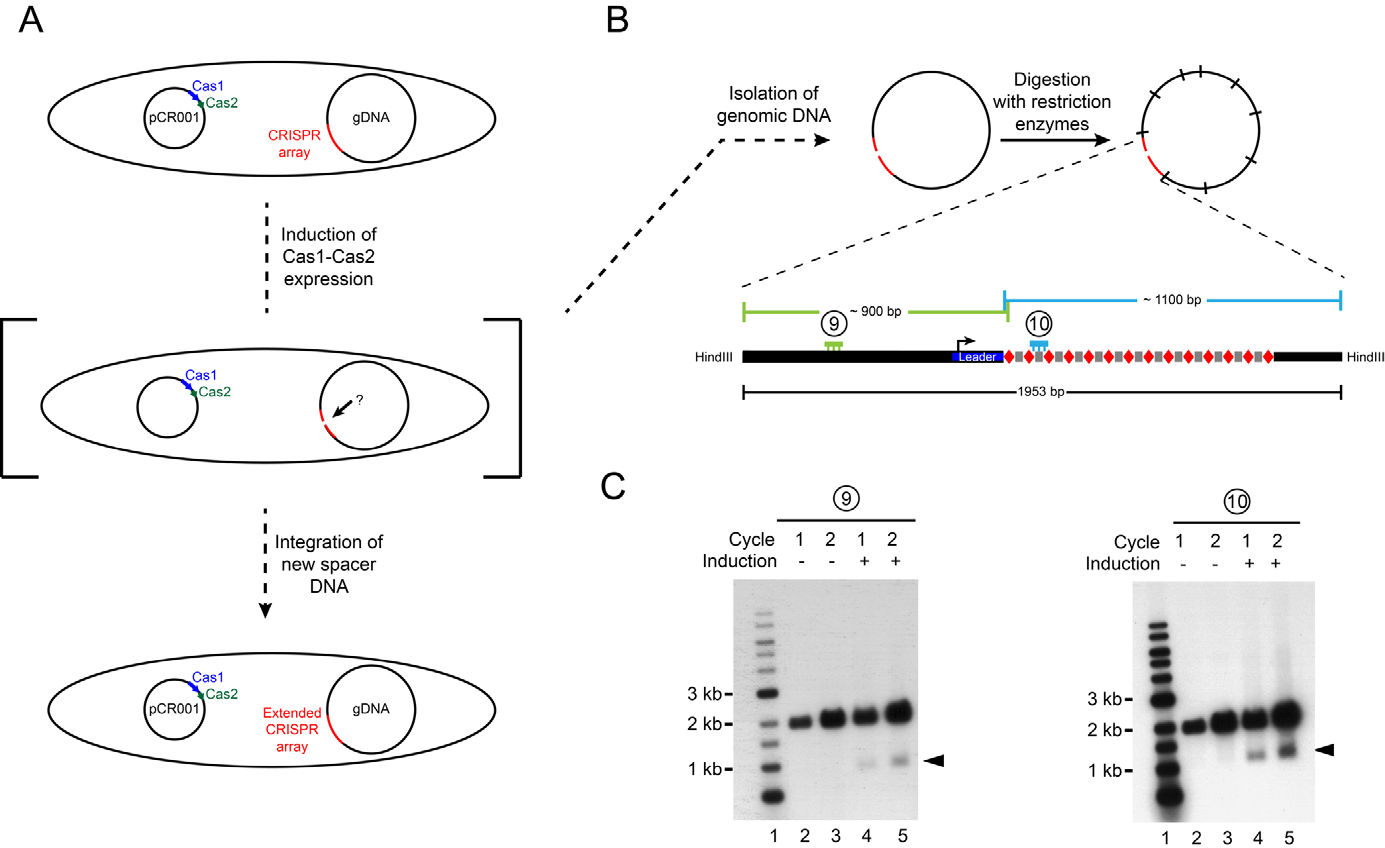
Southern analysis of CRISPR locus after induction of *cas1–cas2* expression. (**A** and **B**) The experimental procedure to test for intermediate states in the course of spacer acquisition. *Escherichia coli* BL21-AI cells were transformed with pCR001 harboring *cas1–cas2* operon as indicated in (A). CRISPR array located on the genomic DNA (gDNA) is colored in red. Genomic DNA was isolated either from cycle 1 or cycle 2 cells with or without induction of plasmid-encoded *cas1–cas2* expression, respectively (see text for details). DNA samples were digested with restriction enzyme HindIII, separated on a 0.7% agarose gel (20 μg DNA in each lane) and blotted onto nylon membrane. (**C**) The fragments with the CRISPR locus were visualized by hybridization of radiolabeled probes complementary to the upstream region of the leader DNA (probe 9, Supplementary Table S4) or to the second spacer of the CRISPR locus (probe 10, Supplementary Table S4). In addition to the native CRISPR locus with a length of 1953 nt, shorter bands were obtained (indicated by arrow heads) when *cas1–cas2* expression was induced (lanes 4 and 5).

Any mechanism for DNA integration has to be initiated by a prior cleavage event at the integration site. Hence, we proposed that in the course of spacer acquisition transient intermediates could exist in which the CRISPR array is locally opened (Figure 1A). Detection and characterization of such intermediate states would allow to address yet unresolved issues, e.g. whether the uptake of spacers is carried out through a staggered cut at the first repeat sequence next to the leader DNA and whether Cas1 and Cas2 do participate at this stage, and which sequences of the leader DNA or repeat determine the specificity for the binding, nicking and ligation by the integration complex.

First, to examine whether the proposed transient intermediates exist and are detectable, we induced the acquisition of new spacers as aforementioned and analyzed the state of CRISPR array by Southern analyses (Figures 1A–C). We isolated genomic DNA from BL21-AI/pCR001 cultures grown for 18 h (cycle 1) or for additional 18 h after inoculation into fresh medium (cycle 2) with (+) or without (−) induction of *cas1–cas2* expression. The isolated total DNA samples were then digested with the restriction enzyme HindIII that led to the fragmentation of the genomic DNA to roughly 515 DNA fragments of different length (ranging from 8 to 63382 bp) (Figure 1B). The fragments were separated on a 0.7% agarose gel and capillary blotted onto nylon membrane. Two radiolabeled oligonucleotides, either complementary to the upstream region of the leader DNA or to the second spacer, were used to visualize the non-template strand (upper strand) of the CRISPR array (Figure 1C). In addition to the fragment with the expected size of 1953 bp that contains the entire CRISPR array, we obtained significant amounts of cleavage products, whose formation depended on the induction of *cas1–cas2* expression (Figure 1C, lanes 4 and 5). The lengths of the bands suggested a cut of the non-template strand, likely in proximity of the leader-repeat junction.

### Characterization of the spacer acquisition intermediates

**Figure 2.**
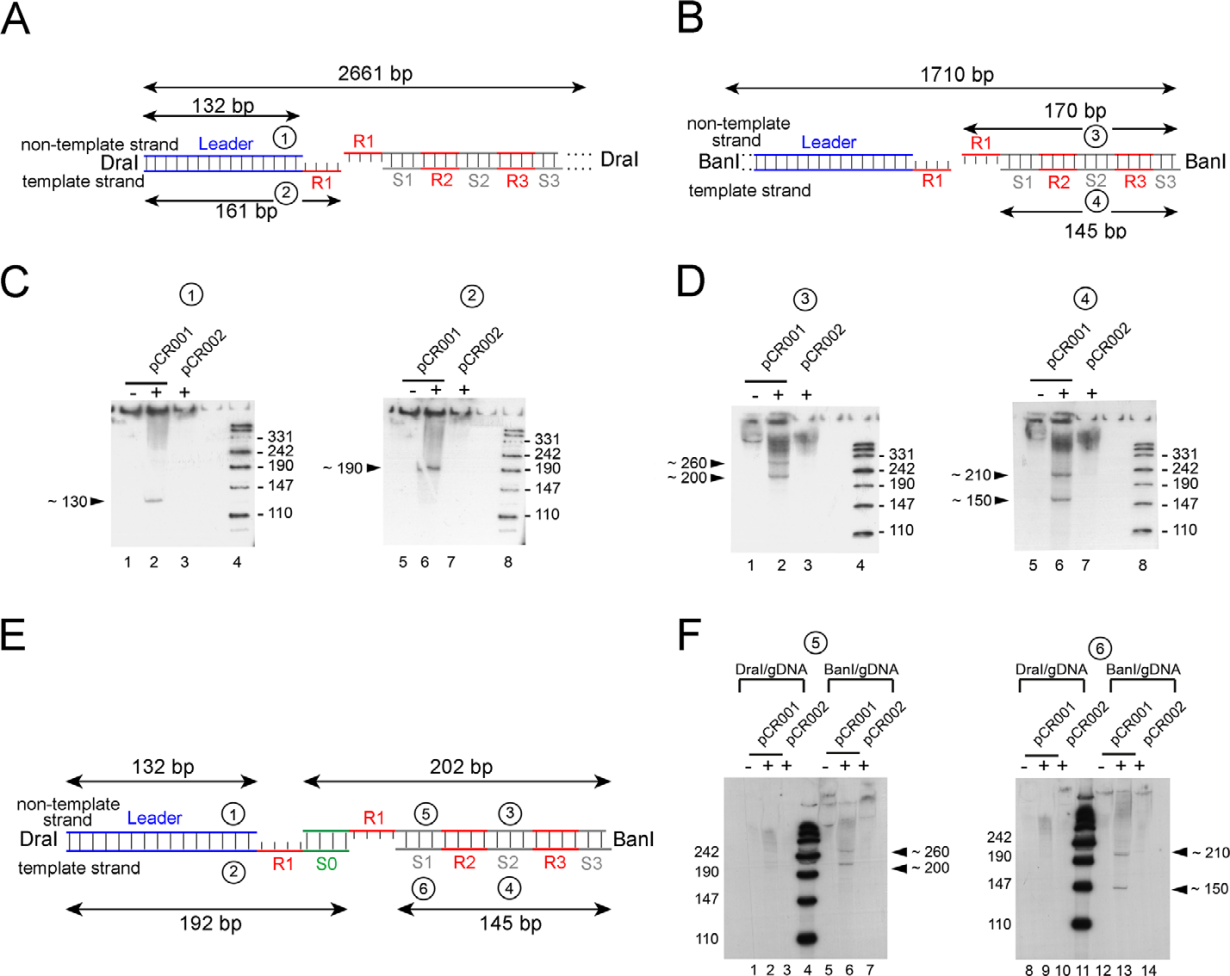
Southern analyses with genomic DNA after induction of spacer integration. Genomic DNAs were prepared from BL21-AI strains harboring either pCR001 (expressing wild-type Cas1 and Cas2 proteins) or pCR002 (expressing D221A Cas1 and wild-type Cas2), grown for 24 h without (−) or with IPTG/arabinose-induction (+). 27.5 μg (in **C** and **D**) or 30 μg (in F) of Dra- or Ban-digested DNA were separated on 10% denaturing polyacrylamide gel and blotted onto nylon membrane. The fragments were visualized by hybridization with radiolabeled oligonucleotides complementary to the non-template or template strand of the leader DNA (probes 1 and 2), the second spacer sequence (probes 3 and 4), or the first spacer (probes 5 and 6). Autoradiograms of the Southern blots obtained with DraI-digested (C, **F**) or BanI-digested genomic DNA (D, F) are shown. Cas1 and Cas2-dependent cleavage products and their lengths are indicated by arrows. (**A** and **B**) The schemes depict the expected lengths of the DNA fragments in the case of a putative Cas1 and Cas2-directed staggered cut at the first repeat sequence. (**E**) Modified model for the intermediates is shown, which considers a concerted cut and ligation of a new spacer DNA (S0, green). R: repeat; S: spacer.

The results presented above indicated that a small proportion of the CRISPR array in the genomic DNA preparation contained a nick within the non-template strand when Cas1 and Cas2 were overexpressed. By subsequent Southern analyses, we carried out a thorough examination of these intermediates. In order to get shorter fragments of the intermediate DNAs, which can be resolved at a higher resolution on 10% denaturing polyacrylamide gels, we digested the genomic DNA preparations either with DraI that cleaves close upstream of the proposed nicking site, or with BanI that cleaves within the third spacer of the CRISPR array (Figure 2A, B; Supplementary Figure S3). In that way, we were able to inspect the upstream (leader region) or downstream part (repeat-spacer cluster) of the integration site more accurately (Figure 2A and B). Furthermore, in order to evaluate the Cas1-specificity of the obtained Southern signals, we introduced a single mutation into the *cas1* gene by site-directed mutagenesis to replace the aspartate of the metal coordinating acidic triad of Cas1 by alanine (pCR002; D221A mutant) (29). Consistent with the study from the Qimron lab (23), this mutation eliminated the acquisition of new spacers (Supplementary Figure S4), and is therefore well suited to correlate the appearance of the intermediates with the activity of Cas1 protein.

We isolated genomic DNA from BL21-AI cultures transformed with either pCR001 (wild-type Cas1 and Cas2) or pCR002 (D221A variant of Cas1 and Cas2) grown for 18 h with or without induction of protein expression. The isolated DNA samples were digested with DraI or BanI, aliquots of the samples were then separated on a 10% denaturing polyacrylamide gel and electroblotted onto nylon membrane. Southern blots were performed with several radiolabeled probes complementary to different regions of the CRISPR array (indicated by the numbers in Figure 2A and B). Overall, the results confirmed the presence of intermediates that were specifically formed when wild-type Cas1 and Cas2 were expressed (Figure 2C and D, lanes 2 and 6). In contrast, no cleavage product was observed under non-induced conditions (lanes 1 and 5) or when Cas1 mutant was expressed (lanes 3 and 7), indicating the Cas1 specificity of observed DNA nicks and the requirement of the metal coordinating center of Cas1. The evaluation of the fragment sizes revealed that the intermediates did not originate from a single cleavage reaction, as indicated in Figure 2A and B, but were likely the result of an integrase reaction in which a staggered cut at the first repeat sequence is accompanied with the ligation of a new spacer DNA at the nicking site. A model of gapped intermediates, as shown in Figure 2E, very well reflects the observed Southern signals. According to this, the non-template strand is nicked at the leader-repeat junction with subsequent joining of the resulting 5′-end of the first repeat with the 3′-end of the incoming spacer (represented by the 130 nt band in lane 2 of Figure 2C and 200 nt band in lane 2 of Figure 2D). Similarly, the template strand is nicked at the first repeat-spacer junction and the 5′-end of the repeat strand is joined to the 3′-end of the new spacer (represented by the 150 nt band in lane 6 of Figure 2D and 190 nt band in lane 6 of Figure 2C). These results were further verified with probes against both strands of the first spacer (Figure 2F). Specific bands were only obtained with BanI-digested genomic DNA (lanes 6 and 13) but not with DraI-digested DNA, consistent with the location of the spacer 1 (S1) downstream of the newly added spacer (S0) and the gaps. The lengths of the bands were, as expected, the same as obtained with probes against the spacer 2 (lanes 2 and 6 in Figure 2D and lanes 6 and 13 in Figure 2F).

**Figure 3.**
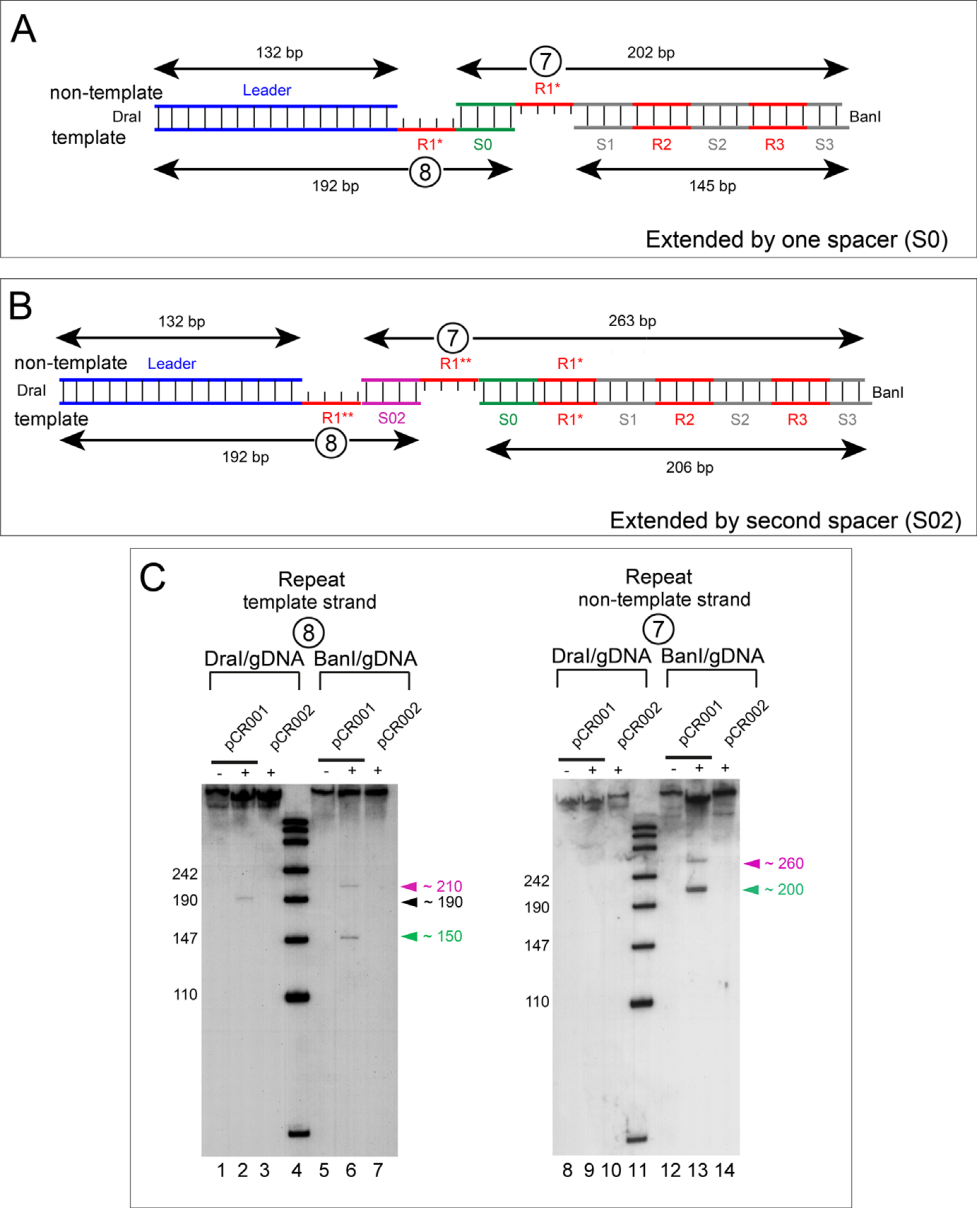
Verification of the gaps. Southern blots were performed as described in Figure 2. Radiolabeled probes complementary to the repeat sequence of the non-template (probe 7) or template strand (probe 8) were hybridized against Dra- or BanI-digested genomic DNA. Schemes of intermediates with one new spacer (S0, green) (**A**) or two new spacer (S0, green and S02, magenta) (**B**). (**C**) Autoradiograms of Southern blots with Dra- (lanes 1–3 and 8–10) and BanI-digested DNA (lanes 5–7 and 12–14) using probe 7 or 8, as indicated.

Next, we verified the gapped DNA structure of the intermediates (Figure 3A). When we used a probe against the repeat sequence of the template strand of DraI-digested DNA, we obtained a specific band again with a length of 190 nt (Figure 3C, lane 2), which fits very well to the result obtained with probes against the leader DNA (Figure 2C, lane 6). However, the same sample hybridized with probes against the non-template strand did not show any apparent band (Figure 3C, lane 9), which is consistent with the lack of the repeat sequence in the non-template strand next to the leader DNA. With BanI-digested genomic DNA and both repeat probes we obtained the same signals as with the spacer-specific oligonucleotides (Figure 3C, lanes 6 and 13).

Additionally, we observed the presence of two signals for both strands of the BanI-digested genomic DNA with all three pairs of probes (against spacer 1, spacer 2 or repeat; Figures 2 and 3). Supported by the sequencing results, which demonstrate an uptake of multiple spacers into the CRISPR array of some single clones (Supplementary Table S1), the slower migrating signals likely arose from a second integration event (Figure 3B). This conclusion is further supported by the fact that the DraI-digested genomic DNA always resulted in one specific signal for both intermediates (132 nt non-template or 192 nt template strand, Figures 2C and 3C), while the BanI-digested sample led to two signals for both strands with all three pairs of probes (Figure 2D and F and 3C). This excludes the possibility of a second cleavage event within a single CRISPR array but rather indicates that each of the two signals with BanI-digested DNA are intermediates of two individual CRISPR arrays, inserting the first (S0, Figure 3A) or a second (S0 and S01, Figure 3B) spacer.

### Role of the repeat sequence in the insertion of new spacer DNA

**Figure 4.**
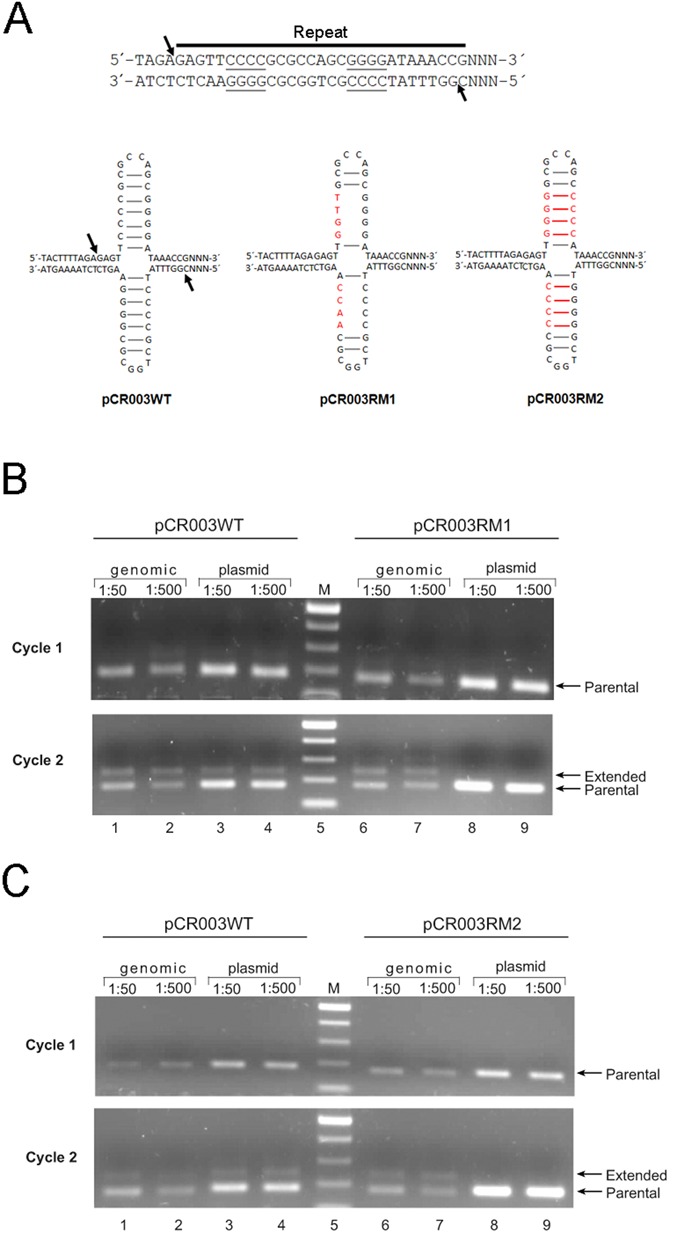
Effects of mutations within the first repeat sequence on spacer acquisition. (**A**) A staggered cut at the first repeat sequence can occur either in a sequence- or structure-specific manner. Putative cruciform structure of the wild-type or mutated repeat-sequences are shown. The arrows indicate the positions of the nicks as deduced from Southern analysis and sequencing of expanded CRISPR arrays. (**B**) 1:50 or 1:500 dilutions of *E. coli* BL21-AI cultures transformed with pCR003WT (lanes 1–4) or pCR003RM1, grown for 18 h after induction of *cas1–cas2* expression (cycle 1), were analyzed by PCR using primers 10 and 15 (amplification of the genomic array) or primers 16 and 17 (amplification of the synthetic array on the plasmids). The PCR products were separated on 1.2% agarose gels. A second round of spacer acquisition was started through transfer of aliquots of the culture into fresh IPTG/arabinose-containing medium and incubation for additional 18 h (cycle 2). The PCR products of the parental CRISPR locus (parental) and those with new spacer DNA (extended) are indicated. (**C**) The same as in (B) but using cells transformed with pCR003RM2 (lanes 6–9).

To rule out that the observed cleavage of the CRISPR array was artificially caused by the overexpression of the Cas1 protein, we tested the DNA sequence specificity by mutational studies. First, we established a ‘plasmid-based CRISPR immunization’ assay, which facilitates the modification of plasmid-located CRISPR sequences by site-directed mutagenesis. We complemented the pCR001 plasmid with a short CRISPR array, consisting of the leader DNA and four repeat sequences, to get the plasmid pCR003WT. The first two repeats of the plasmid-located array are separated by a synthetic spacer sequence, while the others are separated by restriction enzyme sites (Supplementary Table S5). We could previously demonstrate that this synthetic CRISPR array is actively transcribed and the resulting pre-crRNA is processed by the Cascade complex (43). As shown in Supplementary Figure S5, the induction of Cas1 and Cas2 expression resulted in the acquisition of new spacers into the pCR003WT plasmid (see also Figure 4B and C, lanes 3 and 4). Sequencing of the expanded arrays revealed that the new spacers had the expected size, were derived either from genomic DNA or from plasmid DNA, and were flanked by two repeat sequences, demonstrating an accurate integration of new spacers into the plasmid-located CRISPR array (Supplementary Table S2). Moreover, two spacers could be inserted into the CRISPR array on a single plasmid (Supplementary Figure S6, lane 13 and Supplementary Table S2).

The results obtained from the Southern blots presented above indicated a site-specific nicking at the first repeat in which the catalytic center of the Cas1 protein is involved. The recognition of the first repeat could be based on specific interaction of the Cas1 protein with DNA motifs located at the leader-repeat region. Alternatively, or in addition, Cas1 may bind in a DNA structure-specific manner to the CRISPR repeat. As shown by Babu *et al*. (29), Cas1 is able to cleave cruciform DNA structures *in vitro*, which in principle could be formed by the palindromic repeat sequences (Figure 4A). Cruciform DNA structures in palindromic regions are known to be specifically bound by nucleases or integrases during site-specific recombination and integration reactions (44,45). An analogous mechanism could be utilized in the acquisition of new spacers by the CRISPR–Cas system.

To test a DNA structure-specific uptake of new spacers, we designed base mutations that disrupt a potential stem loop structure at the first repeat (pCR003RM1, Figure 4A). In contrast to the pCR003WT plasmid, spacer acquisition assays with pCR003RM1 showed no detectable uptake of new spacers (Figure 4B, lanes 3, 4 and 8, 9). Within the same sample the acquisition into the chromosomal array was intact (lanes 6 and 7), showing that the defect in spacer insertion into the plasmid pCR003RM1 was not caused by indirect effects of the repeat mutations to the expression of the *cas1–cas2* genes located on the same plasmid (Figure 4B, lines 6 and 7). Next, we swapped the positions of the four consecutive C and G bases of the first repeat, which retains the ability of the repeat to adopt cruciform structure but changes its primary sequence (pCR003RM2, Figure 4A). As can be seen in Figure 4C, the insertion of spacer sequences remained inhibited, indicating that a potential cruciform structure of the repeat *per se* is not sufficient for the insertion of new spacers.

### Verification of Cas1 and Cas2-mediated cleavage-ligation reaction using plasmid-based CRISPR array

**Figure 5.**
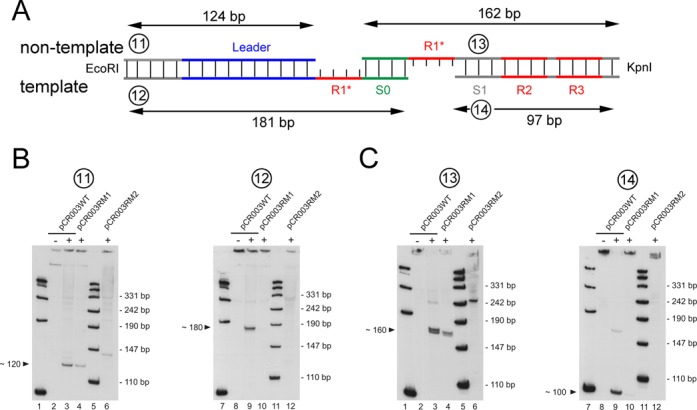
Southern analyses of plasmid-located CRISPR arrays after induction of *cas1–cas2* expression. (**A**) The scheme depicts the expected lengths of DNA fragments in the case of a Cas1 and Cas2-directed cleavage and ligation of new spacer DNA (S0). The plasmid DNA was linearized either with EcoRI (120 bp upstream of the leader-repeat junction) or with KpnI (126 bp downstream of the first repeat). One microgram of each linearized plasmids were separated on 10% denaturing polyacrylamide gels. (**B**) Southern analysis with radiolabeled oligonucleotides (probes 11 and 12, Supplementary Table S4) against the leader DNA of EcoRI linearized plasmids is shown. Lanes 1, 5, 7, 11: length marker; lanes 2, 8: wild-type plasmids isolated from cells without induction of *cas1–cas2* expression; lanes 3, 9: wild-type plasmids isolated from cells with induction of *cas1–cas2* expression; lanes 4, 10: pCR003RM1 plasmids isolated from cells with induction of *cas1–cas2* expression; lanes 6, 12: pCR003RM2 plasmids isolated from cells with induction of *cas1–cas2* expression (**C**) The same as in (B) but with radiolabeled oligonucleotides against the spacer (probes 13 and 14, Supplementary Table S4) of KpnI linearized plasmids.

We examined the formation of the integration intermediates in the plasmid-based CRISPR array, in order to confirm their existence by an independent assay system but also to test the dependence of nicking-ligation reaction on the repeat sequence. To this aim we performed Southern analyses with the plasmids pCR003WT and its variants with mutated repeat sequences. After induction of spacer acquisition and growth of the cells for 18 h at 37°C, we prepared the corresponding plasmid DNAs and linearized them either with EcoRI (cleaving 124 bp upstream of the leader-repeat junction) or with KpnI (cleaving between repeat 3 and 4). Pairs of oligonucleotide were used as probes, which are complementary to both DNA strands upstream of the leader region or to the synthetic spacer at the first position (indicated by the numbers in Figure 5). The lengths of the bands, obtained with the wild-type plasmid confirmed a coupled cleavage-ligation reaction at the first repeat sequence with the same nicking polarity as observed with the genomic DNA. Accordingly, the non-template strand is nicked at the leader-repeat junction (∼120 nt band in lane 3 of Figure 5B), the template strand is nicked at the repeat-spacer junction (∼100 nt band in lane 9 of Figure 5B), and a new spacer DNA is joined to the resulting 5′-ends of the repeat overhangs (∼182 nt band in lane 9 of Figure 5B and ∼160 nt band in line 3 of Figure 5C).

Furthermore, the mutations within the first repeat affected the formation of the intermediates. The nicking-ligation reaction at the non-template strand was considerably reduced in the pCR003RM1 plasmid (compare lanes 3 and 4 in Figure 5B and C), while a nicking-ligation at the template strand was not detectable at all (compare lanes 9 and 10 in Figure 5B and C). The unfavorable effect of repeat mutations on the cleavage-ligation reaction was more prominent with the pCR003RM2 plasmid. A weak signal at the non-template strand revealed a shift of the nicking site to a site more downstream (line 6 in Figure 5B and C), while in the template strand no apparent band was detectable. The altered cleavage patterns obtained with the mutant plasmids further strengthens the specificity of the observed nicking reaction, which thus not only depends on the expression of Cas1 and Cas2 proteins and on the metal-binding center of Cas1 protein, but also on the repeat DNA sequence at the integration site.

In summary, our results provide the first experimental evidence for the involvement of Cas1 protein and its metal-binding center in the staggered cleavage of the CRISPR array at the leader-repeat junction and joining of the incoming spacer in-between of the repeat strands. The absence of any intermediate DNA without newly added spacer DNA at the integration site suggests that the nicking and ligation occur in a concerted manner, corresponding to a classical integrase reaction in which Cas1 and Cas2 proteins catalyze the nucleophilic attack of the 3′-OH groups of the incoming spacer to the 5′-ends of the first repeat in a one-step reaction. Such a mechanism is consistent with the predicted integrase activity of the Cas1 protein (46). During the review process of this manuscript the crystal structure of Cas1–Cas2 complex has been reported (47). The heterotetrameric structure of Cas1–Cas2 complexes with two catalytic centers of Cas1, which are located on the flanks of the heterotetramers, and the preferential binding of the complexes to the leader-repeat sequence are in accordance with a single-step integrase reaction at the first repeat, leading to the gapped intermediates presented in this work. Bioinformatics analyses indicate that in very rare cases the acquisition of new spacers obviously occurs at internal sites within the CRISPR array of *E. coli* (48). We hypothesize that this could be based on erroneous recruitment/binding of Cas1–Cas2 integrase complexes to internal repeat sequences.

## SUPPLEMENTARY DATA

Supplementary Data are available at NAR Online.
